# The Menopause, Its Complications and Treatment

**Published:** 1895-07

**Authors:** N. A. Olive

**Affiliations:** Waco, Texas


For Texas Medical Journal.

### THE MENOPAUSE, ITS COMPLICATIONS AND TREATMENT.

BY N. A. OLIVE, M. D., WACO, TEXAS.

[Read before the Waco Medical Association, May 7, 1895.]

IF IN ANY CLASS of cases a man should be thoroughly equipped with a high order of scientific attainment to assist nature in her wonderful efforts, and not to thwart her magnificent career, it is certainly in this.

The uterus has passed its period of activity; its wonderful accumulation of life has been placed to its credit, it now reluctantly goes out of business. It may be teased, it may be tempted to return to its career of usefulness; but it will not; it is contrary to the natural order of things; it has been transformed,—it is a new being; it has been born again. Woman thus passes from a period of activity, having filled one engagement after another, without feeling any inconvenience from the demands that have been made upon her natural resources. She is a bright, vivacious, healthy, motherly old lady.

She is fond of her offspring, and in her happiest mood when in their society. She loves life, and the fullness thereof, and is loved by everybody. She is now enjoying the delicious fruit of a well spent life. Her labors have been approved by her Creator, and her lot is a happy one.

Thus may we speak of the natural, typical menopause. It is thus with a woman who passed from childhood to womanhood with colors flying; with one who passed through her child bearing period in a state of perfect health; with one who knew little difference between the pregnant and the non-pregnant state, who, in other words, enjoyed uniform health. This condition, it may be said to their credit, more often applies to the lower than to the higher class of American women. The manifold death-dealing fads that are of short life, the miserable corset that seems to have come to stay, the aversion to the pregnant state to the point of feeling it almost a disgrace, the greater fondness for the fickle lights of society than for the warmth of her own fireside, a greater love for freedom to go, see and be seen, than for her own natural offspring, the disposition to dispose of the product of conception at any cost, has led to a train of evil consequences that are to be greatly deplored. And they threaten the ruin of a certain class of our citizenship. The typical American dude may be cited in illustration of this process of deterioration; and should this condition of things continue, the time is not far distant when, by the natural sequel to our riotous living, we will be forced to call monkey brother, instead of grandpa. And I have no doubt that we might now imitate some of the monkey's habits of life with profit to our race.

I do not wish to be understood to mean that this vicious class constitutes a majority of America's women; for no one is more proud than I to boast of her as the embodiment of virtue, the essence of truth, and ever more ready than man to condemn the wrong and do the right thing. But the class alluded to is unfortunately on the increase, and it is within the power of our profession to correct this state of affairs, to a great extent. This fact, coupled with its clinical importance, is my apology for dwelling upon it.

In a healthy woman, not anæmic from previous disease, the nervous system in a state of tranquility, the uterine organs normal, not being in a state of congestion from undue pressure of tight lacing, habitual abortion, with its other serious attendants, as displacements, etc., not having indulged to excess in venereal excitement, having been kept in bed the proper time at confinement for involution to be properly established, having exercised ordinary discretion during the menstrual periods, not having been subject to undue mental or physical strain during the child-bearing period, nor having been the subject of previous disease long neglected, as a rule, you will have very little trouble during the process of utero-genital involution, known as the menopause.

The menses, at this time, may come with some irregularity, but that is not of serious import (unless excessive flow), as the cessation of the function is often normally accomplished in that manner.

If there is excessive menstruation at this time, it is criminal to say it is due to the change of life, and not look for some intelligent cause, and treat it as you would any other time. This is especially important if your patient is suffering any constitutional effects, as anæmia, etc., from the drain upon the system. If there are fibrous, polypoid or fungus growths, curettement and their removal is indicated now, as at other times. And you will get as good results from restoring the uterus to its normal position, in case of displacement, now, as at any other time. If there is malignant disease, it should also be dealt with as at other times, and this condition may be suspected and carefully looked into when an excessive flow occurs during or after the menopause.

Prolonged amenorrhœa from anæmia, constitutional disease, or bad condition of the blood, at an early age, should not be mistaken for the menopause, and the conditions present should be carefully dealt with. After the general health is recovered, and there has been premature atrophy of the organs, the result of previous conditions, amenorrhœa continuing, stimulating local treatment sometimes arrests the atrophic tendency, and often re-establishes the function. For this, electricity and hot douches are important remedies.

If, following or attending the menopause, there exists a train of obscure nervous symptoms, unexplained by other causes, a simple dilatation of the cervix uteri will afford prompt and absolute relief.

The objects to be achieved during the change of life are for the uterus to return to its juvenile condition, all the generative organs to undergo a systematic atrophy, or involution, at the same time to keep up normal excretion by compensatory increased activity of all the excretory organs, as with the skin, kidneys, liver, lungs, bowels, etc. This nature should do; but where it fails, assistance is required, and ordinary judgment and discretion upon the part of physician and patient, with a sufficiently free use of our hot artesian baths, with their various modes of application, will generally accomplish all that is to be desired.

DISCUSSION.

Dr. Hunter: This is a very important subject, and I wish to state here that the doctor has given us a most excellent paper. He touched on one point there that brought to my mind a case that I had a few years ago, of a lady. She was then said to be in the menopause. She had nine physicians to treat her, and the majority of them had told her that she had disease of the brain; in other words, that she was just on the eve of insanity, and the woman was scared to death for fear she would go deranged. Several physicians had told her that there was no doubt but that her brain was affected. She sent for me. I went in and examined her; found about a dozen women in the house at the time, and the woman way lying on the bed, pale as a corpse. She was about forty-nine years of age, I believe she told me.

Looked like she was just about to die. I got the history of the case from the women in the house, and I proposed an examination. I examined her carefully, orally, and then I proposed a vaginal examination and specular at the same time, and they finally agreed to it. I made the examination, and just as quick as I got my finger into the vagina, and got to the os, I found some little concern there somewhat like your thumb, sticking out of the os. I introduced the speculum, and examined and saw it was a polypus just protruding out of the os. I took a pair of dressing forceps and twisted it off. I did not cut it off, because I thought the woman did not have any blood to spare. I twisted it off, and I had a solution of carbolic acid, and I cauterized the stump, and the woman got well. The point is, there are a great many cases of that kind laid to the menopause, that if the physician would examine his case, not take their word for it, and see what is the matter, and relieve it, and the woman will get well in nine cases out of ten.

DR. HALBERT: That was a very interesting paper to me. But I believe the time has passed when an intelligent physician puts his patient off that is having distressing hemorrhages imperiling her life, with assertions that she is suffering from a change of life, and I feel very proud that that time has passed. I do not expect that there is a man in this house, or in this city, and I doubt in our State, that is so far antiquated that when a patient consults him with a hemorrhage, that doesn't tell her that this is a very grave thing and we must look into it. But the time has been when they did that way. Drs. Hunter and Sears saw with me an old lady who was suffering, and upon examination we found not a polypus but a corroding ulcer, which I pronounced malignant. It proved to be a malignant corroding ulcer, and she died. We ought to be impressed with the importance that where a hemorrhage is excessive at all about the change of life, it demands our immediate attention more than any other time in life in the woman's career, and neglect at that time often cuts off the only chance the woman has for a radical operation that will cure her of a malignant trouble. I think that the importance of the time ought to be impressed upon our minds, and we all ought to think of it as men of grave responsibilities, and whenever a woman consults us at that time, it is our bounden duty to examine her, or if she won't submit to an examination, to not treat her another day. I want to take a little issue with the doctor about claiming that all those troubles about the menopause are due to the corset, to some fad, to some mishap, or mismanagement of the case following labor. I believe that some of those derangements of the menopause, such as these nervous flashes, collections of undue amount of fat, and sometimes those hemorrhages are due to no cause that we can find out by any manner of means. It is just one of the nature's ways of doing things that we can not account for. I think sometimes these things will just occur, and we can not help it. I wish he had told us in his paper some means of controlling these nervous flashes, nervous conditions, at the menopause, and following the menopause, sometimes as high as five years, and some means of controlling that same condition when a menopause is produced by an ovariotomy. I must acknowledge that the treatment has been very unsatisfactory to me in a great many cases. I have found that camphor and bromides, and things of that sort, help some, but I have never found that dilating the cervix had relieved them. I never would have thought of doing it if I had found a uterus that seemed to be all right, I never would have thought of dilating the cervix to relieve it.

Dr. King: The paper was an interesting one, and on an interesting subject, and one that we ought to be informed on as soon as possible, because we often are lacking in giving the aid to our patients that we would like to do. At least that has been my lot. I have been unable to give them the relief that I would like to, and especially in regard to these nervous disturbances of which Dr. Halbert spoke. I would like for some gentleman to give us some information in regard to relieving these cases, if they have it.

Dr. Reilly: I would like to report a case that I knew of that would be interesting. I know a lady, 76 years old, that has never ceased menstruating. She has her menstrual period every month while in Texas, but when in her native State, Tennessee, it ceases. She is a very stout, healthy lady every way. I have thought perhaps there was some malignant trouble that was the cause of it.

Dr. Graves: I am very glad to hear the paper discussed. I wish there had been a larger attendance and more full attention to the paper. I think that the question deserves, perhaps, a great deal more attention than we usually give it in the profession, because I feel confident that a number of patients suffer with complications of this kind that we never do anything for. I believe there are severe neuralgias that result from depreciated vitality and the loss of blood at the menstrual periods that go on and on, and perhaps they are given an iron mixture, and the bowels don't move, and they don't get any better, and become almost bed-ridden for a long time. For some of these cases, where constipation has been the rule of life, I believe that a combination of some of the salines, as Epsom salts and tincture of chloride of iron and nux vomica, will do the patient great good, and relieve her of a great many of those symptoms. Some of those cases are relieved by a combination of strychnine and the arsenate of iron. The phosphite of zinc is a preparation that will give some of them, with severe nervous symptoms, a great deal of relief. In regard to the hemorrhages, I fully agree with those who have spoken, and especially with Dr. Halbert, that an examination is necessary, and if fungoid growth or ulcers corroding are found, they should receive proper treatment. In regard to the use of hot baths, I have never seen a case treated on that principle; but I should imagine that perhaps a bath, taken early in the morning, at the low tide of the system, perhaps a cool douche to the spine especially, would be of a great deal of value in building up the nervous system. I believe that ten to twenty grains of the mono-bromide of camphor, given daily, would frequently give them sufficient relief to make them comfortable when they feel these flashes. Another point is the fact that frequently we make a mistake in attributing certain symptoms to the menopause before it is established. A young lady complains of cold flashes, and disordered menstruation, and things of that kind, and, upon examination, you will find high arterial tension, and perhaps uric acid diathesis, and salycilate of soda and alkalies, and combination of iron with the alkalies, will enable that iron to act and build up that patient, and the patient will be relieved. I am one of those who believe a great many of woman's troubles are due to constipation. I would especially call attention to those premature cases of supposed menopause, where the menses are irregular, or where they are absent altogether, and due to something else, and can be corrected.

Dr. King: The nervous troubles, which I have met with very frequently, have not been due to the cause which Dr. Graves suggests; that is, hemorrhage at the menopause. I have met quite a number of them where the monopause has caused the monthly flow to gradually cease, stop a time or two, and come again, and gradually pass off that way, without any undue loss of blood at all, and yet we would have those distressing nervous symptoms, and I think those symptoms were due rather to the lack of the flow of blood than from any flow of blood. The derangement, the change from that monthly flow of blood to no flow of blood is, I imagine, the cause in cases of that kind, Of course, if they have lost unduly, then we would attribute it. in that case, to undue loss of blood, and of course where there is an undue loss of blood, there ought to be no time lost in making an examination, and removing the cause, if possible; and it generally can be done. I think a tonic course of treatment, iron, quinine, strychnine, and such like things, and often the mineral acids, are an advantage, together with an occasional hot bath and a good thorough massage, so as to arouse the latent energies.

DR. GAWNE:—I have nothing of importance to say. My practice has been as indicated in the paper. In those cases I first find out whether or not there is any local trouble, any uterine trouble, and in those cases where these conditions you have described exist, and there is no diseased uterus, my practice has been to act upon the bowels, tax the eliminative process, to see that the bowels are kept open, the skin active, and the kidneys doing their work, together with tonics, and perhaps I have not had as difficult cases as the other gentlemen, but generally I have found that practice very satisfactory. The woman at the close of this period will stand a great deal of action on the bowels, and as a rule, those flashes and headaches and disagreeable feelings will pass off under a course of salines with tonics.

DR. OLIVE:—I am highly pleased with the discussion that has been brought out on this paper, and the importance of such troubles as the uterine polypus has been expanded on. My friend Dr. Hunter, and Dr. Halbert, I see, fully concur in my opinion that all organic troubles, malignant diseases, fibroid or polypoid or any trouble that may exist there, any fungus granulations that may be there should be removed, and that they have their train of disagreeable symptoms that can be met by their removal. Without these things, with apparent menopause, and no ability to detect any organic trouble, and still we have those hot flashes and that nervous condition and so on. In 99 cases out of a hundred, Dr. Gawne strikes the key-note in what he says there in reference to the excretions. If there is no organic trouble; if there is no displacement of the uterus; no fungus or malignant growth to produce nervous conditions, we must look somewhere else. Are the excretory organs performing their functions properly? Now as to that hemorrhage, I want to touch on that. The hemorrhage may exist with no organic conditions there to produce a hemorrhage, but still we have it. The system may become engorged; the skin is not acting properly; the liver and bowels not acting properly, and as we should have an increased excretion of carbon dioxide from the lungs, all these functions are insufficient; there is an abnormal condition there; this matter is not carried off, and what is the result? The good old method of disposing of this excreta is attempted again. The menstrual flow is brought on again. Of course the menstrual flow is an excretion to a certain extent, and it is that that makes this train of nervous symptoms that can not be accounted for, and it is that that often times causes that excessive flow of blood that can not be accounted for by any organic trouble in the uterus. I believe that ninety-nine times out of a hundred, these nervous symptoms, hot flashes, can be accounted for by the fact that the excretory organs are not performing their functions in that exaggerated manner required at this time.

The objects to be achieved, and I can state this upon good authority, during the change of life, are for the utero (genital organs) to return to their juvenile condition, and at the same time to keep up normal excretions (by exaggerated action of the skin), kidneys, liver, lungs, bowels, etc. As to hot water, what is there better than hot baths to keep up a good free action of the skin? What is there better to excite the secretions of the skin? And you will find that in perfect health a person will have clear white healthy looking skin much more so where he bathes often, than if he bathes, say once a week, and the skin will be peforming the functions of excretion much more happily, and the man's life will be prolonged, and his health will be improved by the fact of these baths. These baths I claim are good because of the fact that they excite the excretory organs to a more properly performed function. Now that, in connection with draughts of hot water to produce good free action of the kindeys, to render the contents of the bowels soluble, and make that pass off in a normal condition, and keep all the organs in proper working condition that they may partake equally of the burdens that have been imposed upon them by the cessation of this, one of the most important functions of life. I think that these symptoms can all be accounted for by this state of affairs, and I do think that while we look properly to the uterus for the explanation of certain conditions that do exist at these times, we do not half often enough look to the excretory organs for the share of the trouble that they are responsible for.

